# Comparing Proton Pump Inhibitors and Emerging Acid-Suppressive Therapies in Gastroesophageal Reflux Disease: A Systematic Review

**DOI:** 10.7759/cureus.84311

**Published:** 2025-05-17

**Authors:** Saba Ahmed, Saad Asghar, Maryam Khanzada, Fnu Soxi, Hamesh Gundala Raja, Mehak Gul, Muhammad M Tariq, Momina Abid, Fazal Carpenter

**Affiliations:** 1 Anesthesia and Critical Care, Health Service Executive (HSE) Kerry Community Services, Kerry, IRL; 2 Internal Medicine, Gomal Medical College, Dera Ismail Khan, PAK; 3 Internal Medicine, Liaquat University of Medical and Health Sciences, Jamshoro, PAK; 4 Internal Medicine, Jersey City Medical Center, Jersey City, USA; 5 Internal Medicine, K.A.P. Viswanatham Government Medical College, Tiruchirappalli, IND; 6 Geriatrics, Montefiore Medical Center Wakefield Campus, New York, USA; 7 Internal Medicine, Interfaith Medical Center, New York, USA; 8 Internal Medicine, Foundation University Medical College, Islamabad, PAK; 9 Internal Medicine, University Medical & Dental College, Faisalabad, PAK; 10 Internal Medicine, Nishtar Medical University, Multan, PAK

**Keywords:** fexuprazan, gastroesophageal reflux disease, gerd, mucosal protection, p-cabs, potassium-competitive acid blockers, ppis, proton pump inhibitors, tegoprazan, vonoprazan

## Abstract

This systematic review evaluates the comparative efficacy and safety of proton pump inhibitors (PPIs) versus alternative acid-suppressive strategies in the treatment of gastroesophageal reflux disease (GERD). A comprehensive search was conducted across PubMed, Embase, Scopus, and the Cochrane Central Register of Controlled Trials (CENTRAL), yielding 877 records, of which five randomized controlled trials met the inclusion criteria. These studies examined the effectiveness of newer agents such as potassium-competitive acid blockers (P-CABs), including vonoprazan, fexuprazan, and tegoprazan, as well as the use of mucosal protective agents and varied PPI administration strategies (on-demand vs. continuous). Findings indicated that P-CABs are noninferior or superior to PPIs in terms of symptom relief and mucosal healing, with tegoprazan demonstrating faster symptom control. Long-term use of vonoprazan was associated with higher gastrin levels and histological changes, although no malignant outcomes were reported. The addition of mucosal protective agents to PPI therapy enhanced histological remission and symptom control. On-demand PPI use showed similar outcomes to continuous use, suggesting it may be a viable option for certain patient populations. Overall, the review supports the growing role of individualized and alternative pharmacologic approaches in the effective management of GERD.

## Introduction and background

Gastroesophageal reflux disease (GERD) is a chronic, relapsing condition characterized by the reflux of gastric contents into the esophagus, leading to symptoms such as heartburn, regurgitation, and, in some cases, erosive esophagitis [1]. Affecting a significant portion of the global population, GERD poses a substantial burden on patients' quality of life and the healthcare system. Proton pump inhibitors (PPIs) have long been considered the cornerstone of GERD management due to their ability to suppress gastric acid secretion effectively [2,3]. However, emerging concerns regarding PPI-related adverse effects, incomplete symptom resolution in some patients, and the phenomenon of rebound acid hypersecretion have prompted a re-evaluation of their long-term use [4].

Recent years have witnessed the development and increasing clinical application of potassium-competitive acid blockers (P-CABs), such as vonoprazan, fexuprazan, and tegoprazan, which offer a different mechanism of acid suppression by directly inhibiting the H+/K+-ATPase in a potassium-competitive, reversible manner [5,6]. These newer agents have demonstrated faster onset of action, longer duration of acid suppression, and potential superiority in healing erosive esophagitis, especially in PPI-refractory cases [7]. Additionally, the optimal mode of PPI administration, whether on-demand or continuous, remains an important consideration in personalizing GERD management. As clinical trials increasingly compare these novel therapies with traditional PPIs, a comprehensive synthesis of their efficacy and safety profiles is both timely and essential to guide evidence-based clinical decision-making.

This systematic review aims to evaluate the comparative efficacy and safety of PPIs versus novel acid-suppressive therapies, including P-CABs and modified PPI administration strategies, in the treatment of GERD [8]. The population of interest includes adult patients diagnosed with GERD, with or without erosive esophagitis. The interventions assessed comprise standard PPIs such as rabeprazole and esomeprazole, administered either on-demand or continuously. The comparison group includes newer agents such as vonoprazan, fexuprazan, and tegoprazan, as well as mucosal protective therapies used either alone or in combination with PPIs. The primary outcomes focus on the resolution of GERD symptoms, healing of erosive esophagitis, improvement in quality of life, and incidence of adverse events. Through this Patient, Intervention, Comparison, and Outcome (PICO) framework, the review seeks to clarify whether novel approaches to acid suppression provide superior or equivalent clinical outcomes compared to conventional PPI therapy in the modern management of GERD.

## Review

Materials and methods

Search Strategy

The search strategy for this systematic review was designed to comprehensively identify relevant clinical trials comparing the efficacy of PPIs with alternative acid-suppressive therapies in the treatment of GERD. A targeted and structured search was conducted across multiple databases, including PubMed, Embase, Scopus, and the Cochrane Central Register of Controlled Trials (CENTRAL), using a combination of Medical Subject Headings (MeSH) and free-text keywords such as "GERD", "gastroesophageal reflux", "proton pump inhibitors", "H2-receptor antagonists", "potassium-competitive acid blockers", "P-CABs", and "clinical trials". Filters were applied to limit the search to human studies, clinical trials, and publications from the last one year, ensuring the inclusion of the most up-to-date and relevant literature. The review adhered to the Preferred Reporting Items for Systematic Reviews and Meta-Analyses (PRISMA) guidelines [9], and the study selection process included the independent screening of titles and abstracts, followed by full-text reviews of eligible studies. A total of five high-quality randomized controlled trials (RCTs) were selected for final inclusion based on predefined inclusion and exclusion criteria. Studies were excluded if they did not directly assess pharmacologic interventions for GERD, focused on unrelated reflux conditions, or were conducted in populations outside the scope of this review.

Eligibility Criteria

The eligibility criteria for this systematic review were established using the PICO framework to ensure the inclusion of high-quality, relevant studies focused on evaluating the comparative efficacy of PPIs and alternative pharmacological therapies in the treatment of GERD. Eligible studies included RCTs published within the last one year, involving adult patients (≥18 years) diagnosed with GERD, with or without erosive esophagitis. Interventions of interest included PPIs (administered either continuously or on-demand), P-CABs such as vonoprazan, fexuprazan, and tegoprazan, and combination therapies involving PPIs and mucosal protective agents. Studies were required to report clinical outcomes related to symptom relief, mucosal healing, histological improvement, quality of life, or safety parameters.

Exclusion criteria included non-randomized studies, reviews, case reports, editorials, studies involving pediatric populations, or those evaluating non-pharmacologic interventions such as surgical procedures, implants, or traditional herbal formulations not used in mainstream medical practice. Additionally, trials focusing on conditions distinct from GERD, such as laryngopharyngeal reflux or esophageal motility disorders, were excluded to maintain focus. Only articles published in English and available in full-text were considered. This approach ensured that the included studies provided robust and recent clinical evidence directly relevant to the comparative effectiveness of acid-suppressive therapies in managing GERD.

Data Extraction

Data extraction was performed systematically from each of the included RCTs using a standardized data collection template. Key study characteristics extracted included author and year of publication, study design, sample size, population demographics, intervention and comparator details, duration of therapy, and primary reported outcomes. Emphasis was placed on capturing data related to clinical efficacy, such as symptom relief, healing of erosive esophagitis, quality of life measures, and histological changes, as well as any reported safety outcomes. The extraction process was conducted manually, ensuring consistency and accuracy across studies. Discrepancies were resolved by re-evaluating the original study reports. The extracted data were then tabulated to allow for the qualitative synthesis and comparison of findings across different interventions.

Data Analysis and Synthesis

Data analysis and synthesis were conducted using a qualitative, narrative approach due to the heterogeneity of interventions, outcome measures, and study designs across the included trials. The extracted data were systematically organized into comparative tables to highlight similarities and differences in clinical efficacy, safety profiles, and treatment duration among the evaluated therapies. Given the variation in dosing regimens, endpoints, and reporting formats, a meta-analysis was not feasible. Instead, key findings were synthesized to identify consistent trends, such as the noninferiority of P-CABs to PPIs in healing erosive esophagitis, the faster symptom relief observed with certain agents, and the added benefit of mucosal protective agents when combined with PPIs. The synthesis aimed to provide a balanced interpretation of the evidence while accounting for study quality, risk of bias, and clinical relevance.

Results

Study Selection Process

**Figure 1:**
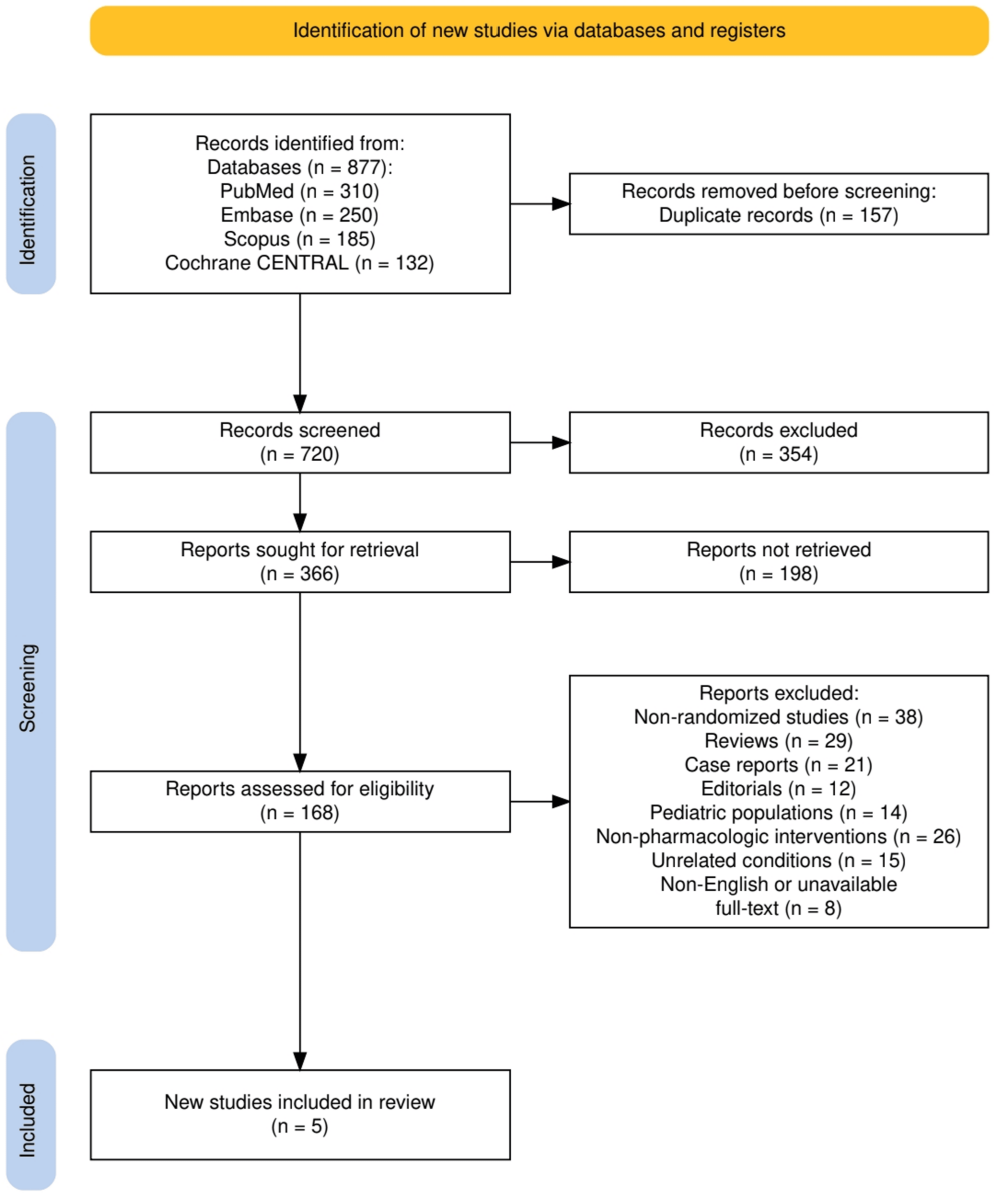
The PRISMA flowchart represents the study selection process. PRISMA: Preferred Reporting Items for Systematic Reviews and Meta-Analyses; Cochrane CENTRAL: Cochrane Central Register of Controlled Trials

The study selection process is summarized in Figure 1, which outlines the application of the PRISMA 2020 flow diagram. A total of 877 records were identified through database searches, including PubMed (310), Embase (250), Scopus (185), and Cochrane CENTRAL (132). After the removal of 157 duplicate records, 720 records remained for title and abstract screening. Of these, 354 records were excluded for not meeting the initial eligibility criteria. The remaining 366 reports were sought for full-text retrieval, but 198 could not be retrieved, leaving 168 reports for detailed eligibility assessment. Following full-text review, 163 studies were excluded due to the following reasons: non-randomized studies, reviews, case reports, editorials, pediatric-focused research, non-pharmacologic interventions, unrelated reflux conditions, or lack of accessible full texts in English. Ultimately, five RCTs that met all inclusion criteria were included in the final systematic review.

Characteristics of the Selected Studies

The characteristics of the included studies are summarized in Table 1. All five studies were RCTs, varying in design from open-label to double-blind and conducted across diverse clinical settings, including multicenter trials and real-world primary care environments. Sample sizes ranged from 60 to 488 participants, with populations comprising adult patients diagnosed with GERD, including those with healed erosive esophagitis or prior response to acid-suppressive therapy. Interventions evaluated included both continuous and on-demand regimens of PPIs, newer P-CABs such as vonoprazan, fexuprazan, and tegoprazan, as well as a combination of PPI with a mucosal protective agent. The duration of treatment varied from short-term trials of 4-8 weeks to a long-term maintenance study extending up to 260 weeks. Reported outcomes focused on mucosal healing, symptom control, histological improvement, patient satisfaction, quality of life, and treatment-emergent adverse events, providing a comprehensive view of the comparative efficacy and safety of various GERD treatment strategies.

**Table 1:** Summary of clinical trials comparing P-CABs versus PPIs in the treatment of GERD. P-CAB: potassium-competitive acid blocker; PPI: proton pump inhibitor; GERD: gastroesophageal reflux disease; EE: erosive esophagitis; HRQL: health-related quality of life; MPA: mucosal protective agent; FAS: full analysis set; RCT: randomized controlled trial; ECL: enterochromaffin-like; NETs: neuroendocrine tumors; PPS: per-protocol set

Study (author, year)	Study design	Sample size	Population characteristics	Intervention	Comparator	Duration	Outcome
Uemura et al., 2025 [10]	Phase IV RCT, open-label	N=208 (maintenance: vonoprazan n=139; lansoprazole n=69)	Japanese adults with healed erosive esophagitis after initial PPI/P-CAB therapy	Vonoprazan 10 mg daily (after 20 mg induction)	Lansoprazole 15 mg daily (after 30 mg induction)	260 weeks (5 years)	Incidence of gastric mucosal changes (parietal cell hyperplasia, foveolar hyperplasia, ECL-cell hyperplasia, G-cell hyperplasia); median serum gastrin levels; absence of malignant epithelial changes or gastric NETs
Zhuang et al., 2024 [11]	Phase III RCT, double-blind, multicenter	N=332 (FAS), 311 (PPS)	Adults with endoscopically confirmed erosive esophagitis	Fexuprazan 40 mg once daily	Esomeprazole 40 mg once daily	4-8 weeks	Noninferiority of EE healing rates at 8 weeks (FAS: 88.5% vs. 89%; PPS: 97.3% vs. 97.9%); similar symptom response, GERD-HRQL improvement, and adverse event rates
Andreasson et al., 2024 [12]	Pragmatic RCT, real-world, multicenter	N=488 enrolled (360 completed follow-up)	Adult GERD patients in primary care (median age 51, 58% female)	On-demand PPI prescription	Continuous PPI prescription	8 weeks	Both strategies improved reflux symptoms and quality of life; no significant difference between groups; increased PPI use associated with improved outcomes
Kang et al., 2025 [13]	RCT, multicenter	N=69 (completed)	GERD patients with symptom improvement on prior acid-suppressive therapy	On-demand tegoprazan 50 mg	On-demand esomeprazole 20 mg	8 weeks	No significant difference in patient satisfaction; tegoprazan showed faster symptom relief (26.2% vs. 16.1% improved within 30 minutes); no serious adverse events
Bordin et al., 2024 [14]	RCT, open-label, prospective	N=60	Adults with erosive GERD (mean age 41.5 years, 5-year disease duration)	PPI+MPA	PPI monotherapy	4 weeks	Combination therapy led to greater symptom relief, more pronounced endoscopic improvement, and improved histological remission (tight junction restoration, reduced inflammation) compared to PPI alone

Quality Assessment

The quality assessment of the included studies, summarized in Table 2, was conducted using the Cochrane Risk of Bias 2 (RoB 2) tool. Two studies were judged to have an overall low risk of bias, reflecting strong methodological rigor with well-executed randomization, complete outcome reporting, and consistent adherence to intended interventions. These included the trials evaluating fexuprazan and tegoprazan, both of which were double-blind and had no significant methodological limitations. Three studies were rated as having some concerns, primarily due to open-label designs or issues related to adherence and incomplete follow-up. The long-term vonoprazan trial and the study assessing mucosal protection were open-label, raising concerns about potential performance or detection bias. The pragmatic real-world study on on-demand versus continuous PPI use had minor concerns related to treatment adherence variability and participant dropouts at follow-up. Despite these limitations, all included studies demonstrated generally sound methodological frameworks, allowing for a reasonable level of confidence in the synthesized findings.

**Table 2:** Quality assessment of the included studies.

Study (author, year)	Randomization process	Deviations from intended interventions	Missing outcome data	Measurement of outcome	Selection of reported results	Overall risk of bias
Uemura et al., 2025 [10]	Some concerns (open-label)	Low risk	Low risk	Low risk	Low risk	Some concerns
Zhuang et al., 2024 [11]	Low risk	Low risk	Low risk	Low risk	Low risk	Low risk
Andreasson et al., 2024 [12]	Low risk	Some concerns (real-world variability in adherence)	Some concerns (dropouts at follow-up)	Low risk	Low risk	Some concerns
Kang et al., 2025 [13]	Low risk	Low risk	Low risk	Low risk	Low risk	Low risk
Bordin et al., 2024 [14]	Some concerns (open-label)	Low risk	Low risk	Low risk	Some concerns (limited reporting of analysis plans)	Some concerns

Discussion

The findings from the five RCTs included in this review collectively suggest that emerging acid-suppressive therapies and individualized PPI strategies may offer comparable and in some instances superior, clinical outcomes to conventional continuous PPI monotherapy in managing GERD. Uemura et al. [10] reported that long-term vonoprazan maintenance therapy was associated with histological changes such as parietal and foveolar cell hyperplasia, although no malignant transformation was observed during the study period, highlighting a potential safety concern that warrants longer-term surveillance beyond the study's follow-up duration. Zhuang et al. [11] demonstrated the noninferiority of fexuprazan to esomeprazole in mucosal healing, but the study showed slightly lower healing rates in the full analysis set (FAS) population, which could indicate variability in drug response depending on patient subgroups. In contrast, Andreasson et al. [12] found no significant difference in symptom control or quality of life between on-demand and continuous PPI use in a real-world primary care setting, although this finding may be influenced by differing baseline severity levels and adherence patterns. Kang et al. [13] observed more rapid symptom relief with tegoprazan than esomeprazole, yet both drugs showed similar overall patient satisfaction, suggesting that speed of onset may not universally translate to higher patient-perceived efficacy. Finally, the study by Bordin et al. [14] showed notable benefits with combination therapy involving PPIs and mucosal protective agents, although the small sample size and short duration limit generalizability. These findings, taken together, underscore the potential of tailoring GERD treatment based on individual clinical characteristics, but also highlight the need to interpret results within the context of study design, sample characteristics, and endpoint definitions.

The overall conclusions of this review are broadly consistent with prior literature supporting the utility of P-CABs like vonoprazan, fexuprazan, and tegoprazan as viable alternatives to PPIs in GERD management [15]. The faster symptom relief observed with tegoprazan in Kang et al. [13] reinforces earlier pharmacodynamic studies indicating more rapid acid suppression with P-CABs [6]. However, this finding contrasts with the results of Andreasson et al. [12], where no significant difference was observed between intermittent and continuous PPI therapy, highlighting a key variation in treatment outcomes that may depend on disease chronicity, patient expectations, and healthcare settings. The noninferiority of fexuprazan to esomeprazole seen in Zhuang et al. [11] was based on comparable rates of erosive esophagitis healing, though effect sizes varied slightly between full analysis and per-protocol populations, reflecting the impact of adherence and dropout rates on treatment evaluation. The combination approach evaluated by Bordin et al. [14], while promising, employed distinct outcome definitions, such as histological remission via tight junction restoration, that are not standardized across trials, thereby complicating direct comparisons. Collectively, these variations in outcome definitions, patient populations, and follow-up durations highlight a degree of heterogeneity that should temper the direct comparability of study results and underscore the importance of nuanced interpretation in clinical decision-making.

Across the included trials, interventions involving newer acid-suppressive agents or enhanced therapeutic strategies such as mucosal protection and on-demand dosing showed comparable or improved efficacy when measured against standard PPI therapy. Vonoprazan, as reported in the long-term study by Uemura et al. [10], maintained mucosal healing but resulted in higher rates of cellular hyperplasia and elevated gastrin levels compared to lansoprazole, raising important questions about long-term biological effects. Fexuprazan was shown to be noninferior to esomeprazole in terms of healing rates and symptom control, although modest differences between FAS and per-protocol set may reflect heterogeneity in adherence or baseline disease severity. Tegoprazan demonstrated a more rapid onset of action than esomeprazole [16,17], offering a practical advantage for patients seeking immediate relief; however, similar levels of patient satisfaction across groups suggest that speed of relief alone may not fully determine treatment success. Furthermore, the use of a mucosal protective agent in combination with PPI significantly enhanced both clinical and histological outcomes, suggesting a synergistic effect [18], though the use of non-standardized histologic markers, such as tight junction integrity, limits comparability with other trials. On-demand PPI usage yielded comparable symptom control to continuous therapy, but definitions of symptom resolution, variability in dosing, and short follow-up durations introduce interpretative limitations that merit caution.

These findings have several important clinical implications. First, they support a more individualized approach to GERD management, allowing clinicians to tailor therapy based on patient preferences, symptom patterns, and treatment response [19,20]. For patients requiring rapid symptom relief or who are refractory to standard PPIs, P-CABs may offer meaningful therapeutic advantages. The safety and efficacy of long-term vonoprazan use, while encouraging, highlight the need for careful monitoring, such as periodic serum gastrin level assessments and endoscopic surveillance to detect potential mucosal or histological changes, given its association with hypergastrinemia and cellular hyperplasia [21]. The demonstration that on-demand PPI use can be as effective as continuous therapy may also help reduce unnecessary medication burden and potential long-term adverse effects in select patient populations. However, the clinical success of this strategy appears closely tied to symptom frequency and patient adherence, both of which varied across the included studies. Additionally, the heterogeneity in endpoints, including variations in how symptom relief was defined, the duration of follow-up, and outcome measurement tools, limits direct comparability and emphasizes the need for standardization in future trials. Finally, the addition of mucosal protective agents could be considered in patients with erosive esophagitis who do not fully respond to acid suppression alone. Together, these insights may inform future guideline updates and enhance shared decision-making in GERD treatment planning [22].

The observed effects across the included studies can be attributed to distinct pharmacological mechanisms and tailored treatment strategies that target both acid suppression and mucosal defense. P-CABs such as vonoprazan, fexuprazan, and tegoprazan act by reversibly inhibiting the gastric H+/K+-ATPase in a potassium-competitive manner, resulting in a faster onset and more sustained acid suppression compared to the irreversible mechanism of action and delayed onset of traditional PPIs [23]. This mechanistic advantage likely explains the more rapid symptom relief associated with tegoprazan and the comparable healing rates achieved with fexuprazan. However, this potent and prolonged acid suppression may also underlie the elevated serum gastrin levels and histological alterations observed with long-term vonoprazan use, such as parietal and enterochromaffin-like cell hyperplasia, due to feedback-driven hypergastrinemia. The enhanced outcomes seen with the combination of PPIs and mucosal protective agents, as reported by Bordin et al. [14], may reflect a complementary mechanism, strengthening epithelial barrier integrity and modulating tight junction proteins, thereby improving both clinical symptoms and histological healing. These findings reinforce the importance of addressing both acid suppression and mucosal vulnerability in optimizing GERD therapy, particularly for patients with erosive disease or incomplete response to PPIs.

The methodological strengths of the included studies lend credibility to the synthesized evidence. All five trials were RCTs, the gold standard in clinical research, and several employed rigorous designs, including double-blinding and multicenter implementation. Notably, Zhuang et al. [11] and Andreasson et al. [12] enrolled relatively large and diverse patient populations, improving the external validity of their findings. The inclusion of both short-term efficacy trials and long-term maintenance studies offers a comprehensive perspective on treatment outcomes over varying durations. Nonetheless, certain limitations must be acknowledged. The open-label design of Uemura et al. [10] and Bordin et al. [14] introduces the risk of performance and detection bias, potentially influencing subjective outcomes such as symptom reporting. Small sample sizes in studies like Kang et al. [13] and Bordin et al. [14] reduce statistical power and limit the generalizability of their findings. Most importantly, substantial heterogeneity was noted in outcome definitions, ranging from symptom scales and histologic criteria to patient satisfaction metrics, as well as differences in follow-up duration, intervention dosing, and population characteristics. These inconsistencies complicate direct comparisons and may obscure subtle differences in efficacy or safety between interventions.

The decision to restrict inclusion to studies published within the last one year was driven by the rapidly evolving nature of GERD management, particularly the emergence of novel acid-suppressive agents such as P-CABs and updated administration strategies like on-demand PPI use and mucosal protective adjuncts. These therapeutic approaches have gained prominence only recently, with several high-quality RCTs published in the past year reflecting current clinical practice and pharmacologic advancements. Limiting the scope to recent literature allowed us to capture a focused and contemporary snapshot of evidence, ensuring relevance to current treatment paradigms and avoiding redundancy with previous systematic reviews. Furthermore, this time-bound approach minimizes the inclusion of outdated or superseded data, thereby strengthening the practical utility and timeliness of our findings for clinicians and researchers alike.

Based on current evidence, clinicians should consider integrating emerging acid-suppressive therapies, such as P-CABs, into routine GERD management, particularly for patients who require rapid symptom relief or exhibit partial response to conventional PPIs [24,25]. The option of on-demand PPI therapy appears viable for select patients with intermittent or mild symptoms, potentially reducing unnecessary medication exposure and associated risks. Combination therapy involving PPIs and mucosal protective agents may be particularly beneficial in patients with erosive esophagitis or histologic evidence of impaired mucosal integrity. However, these recommendations must be tempered by the limitations in current evidence, particularly the long-term safety profile of P-CABs, such as vonoprazan, in light of observed hypergastrinemia and mucosal proliferation. Future research should prioritize well-powered, multicenter RCTs directly comparing different P-CABs with established PPIs across standardized outcome frameworks. Moreover, mechanistic studies investigating the molecular basis of mucosal healing and exploring predictive biomarkers could pave the way for precision-guided therapy in GERD. Such advances may ultimately refine treatment paradigms and enhance individualized care strategies in this common but heterogenous gastrointestinal disorder.

## Conclusions

The evidence from recent RCTs supports the efficacy and safety of emerging acid-suppressive therapies, including P-CABs and mucosal protective strategies, as viable alternatives or adjuncts to traditional PPI therapy in the management of GERD. While P-CABs such as fexuprazan and tegoprazan demonstrate comparable or faster symptomatic relief than PPIs, and combination regimens with mucosal protectants offer enhanced histological healing, the long-term safety profile, particularly regarding cellular changes with agents like vonoprazan, warrants continued surveillance. On-demand PPI therapy remains a practical approach for appropriately selected patients, providing symptom control without the need for continuous dosing. Taken together, these findings highlight the evolving landscape of GERD treatment and underscore the importance of individualized, evidence-based therapeutic decisions grounded in both clinical efficacy and patient-centered considerations.
